# Outcomes of medical dissolution for suspected struvite uroliths in dogs using a therapeutic multipurpose urinary diet and antimicrobial therapy

**DOI:** 10.1093/jvimsj/aalaf061

**Published:** 2026-01-21

**Authors:** Alyssa R Toillion, Selena Gilyot, Jody P Lulich, Eva Furrow

**Affiliations:** Hill’s Pet Nutrition, Inc., Topeka, KS 66601, United States; Hill’s Pet Nutrition, Inc., Topeka, KS 66601, United States; Department of Veterinary Clinical Sciences, University of Minnesota College of Veterinary Medicine, St. Paul, MN 55108, United States; Department of Veterinary Clinical Sciences, University of Minnesota College of Veterinary Medicine, St. Paul, MN 55108, United States

**Keywords:** canine, stones, cystoliths, urinary tract infection, magnesium ammonium phosphate

## Abstract

**Background:**

The consensus recommendation is to medically dissolve suspected struvite uroliths in dogs. However, more data is needed on outcomes for protocols using therapeutic multipurpose urinary foods formulated for both struvite urolith dissolution and calcium oxalate urolith prevention.

**Hypothesis/Objectives:**

To describe outcomes for medical dissolution of suspected struvite uroliths in dogs using a therapeutic multipurpose urinary food and concurrent or recent antimicrobial therapy.

**Animals:**

Thirteen client-owned dogs with suspected struvite uroliths.

**Methods:**

Single-institution, retrospective case series (observational) of dogs fed a therapeutic multipurpose urinary food for dissolution of suspected struvite uroliths. Dogs had to be treated with antimicrobials concurrently or have an absence of bacteriuria on urine microbial culture after recently completed antimicrobial therapy. Follow-up abdominal imaging was required. Patient and urolith characteristics, dissolution outcomes, and complications were summarized.

**Results:**

Complete urolith dissolution occurred in 9 (8 with cystoliths and 1 with nephroliths) of 13 dogs included in the study. Mean time until documentation of complete dissolution was 71 ± 52 days. Eight of the dogs with complete dissolution had bacteriuria less than 60 days prior to urolith diagnosis. Mean duration of antimicrobial therapy for dogs with successful dissolution was 66 ± 49 days. All dogs without complete dissolution had uroliths with non-struvite shells (composed of 80%-100% calcium). No dog developed a urethral obstruction during the dissolution trial.

**Conclusions and clinical importance:**

Struvite uroliths can be effectively dissolved in dogs with a therapeutic multipurpose urinary food and antimicrobial therapy. A non-struvite urolith layer is a common cause of unsuccessful dissolution.

## Introduction

Struvite (magnesium ammonium phosphate) is the most common mineral composition of uroliths in dogs.^1^ Urease-producing bacteria, such as *Staphylococcus* species, are commonly detected in the urine of dogs with struvite uroliths.^2^ Urease-producing bacteria promote struvite formation through the production of ammonium and alkaline urine.^3^ The American College of Veterinary Internal Medicine consensus recommendation is to medically dissolve struvite uroliths with few exceptions.^4^ However, details are not provided on specific foods or protocols to use for medical dissolution.

A canine food formulated for struvite dissolution (Hill’s Prescription Diet s/d Canine Urinary Care, Hill’s Pet Nutrition, Inc.) is effective for medical dissolution of suspected struvite uroliths, especially in combination with antimicrobial therapy.^5^ However, the manufacturer recently discontinued production of this food. Therapeutic multipurpose urinary foods are an alternative option formulated for both struvite urolith dissolution and calcium oxalate (CaOx) urolith prevention by reducing estimates of struvite and CaOx supersaturation.^6–8^ One such food dissolved suspected struvite cystoliths in 5/10 dogs when fed in combination with antimicrobial therapy.^9^ Combination dietary and antimicrobial therapy can also dissolve struvite nephroliths in dogs.^10^ A different protocol using antimicrobial therapy with a urinary acidifier instead of a therapeutic food dissolved suspected struvite cystoliths in 8/11 dogs.^11^ While these studies show that struvite dissolution can be achieved using different protocols, more data is needed on the efficacy and outcomes of medical dissolution of suspected struvite uroliths in dogs using multipurpose therapeutic urinary foods.

The objective of this retrospective study was to describe outcomes for dogs that underwent a medical dissolution trial for suspected struvite uroliths using a therapeutic multipurpose urinary food and concurrent antimicrobial therapy or recent antimicrobial therapy with confirmed absence of bacteriuria with urine microbial culture. We hypothesized that this protocol would be effective for struvite urolith dissolution. We further hypothesized that the primary cause for dissolution failure would be a non-struvite composition, as observed in prior studies.^5^^,^^9^^,^^11^

## Materials and methods

### Case identification

This retrospective case series (observational) was conducted at the University of Minnesota Veterinary Medical Center (UMN VMC). Electronic medical records were searched for dogs seen between January 2020 and July 2024 with the keywords “struvite” and “c/d” in the client discharge letter. Dogs were included if they had medical documentation of uroliths with suspected struvite composition and underwent a dissolution trial that consisted of feeding a therapeutic multipurpose urinary food (Hill’s Prescription Diet c/d Multicare dog food, Hill’s Pet Nutrition, Inc.). Dogs were also required to have received antimicrobial therapy for treatment of suspected or confirmed urinary tract infection, have no bacterial growth on urine microbial culture after recent antimicrobial therapy (completed less than 30 days prior to the trial start date), or both. Dogs were excluded if there was no follow-up abdominal imaging available from the UMN VMC or the referring veterinary clinic. Dogs were also excluded if they were receiving a urinary acidifier, were being fed a combination of different therapeutic urinary foods at the start of the trial, or were switched to a different therapeutic urinary food before the end of the trial.

### Data collection

The baseline visit was designated as the visit after diagnosis of suspected struvite uroliths but preceding commencement of the dissolution trial. The start of the dissolution trial was the first day after urolith diagnosis that the urinary food was fed and either antimicrobial therapy was administered or absence of bacteriuria was confirmed with urine microbial culture. Follow-up visits are referred to relative to the start of the dissolution trial. Sex, age, breed, and weight were obtained from the baseline visit. For the baseline visit and subsequent visits, clinical history was extracted from the medical records and included the clinical service(s) that initiated the trial, clinical signs, food type (dry or canned), and treats given during the dissolution trial. Antimicrobial therapy prescribed up to 30 days prior to or during the dissolution trial was recorded.

Urine collection and analysis methods were recorded. For urinalyses performed at the UMN VMC Clinical Pathology Laboratory, urine was stored at room temperature (approximately 22 °C) and analyzed within 6 h of collection. Urine pH was measured with a dipstick, and sediment was manually reviewed by laboratory technicians. Urinalysis results were reviewed to obtain the urine specific gravity (USG), dipstick pH (<7 = acidic, 7 = neutral, >7 = acidic), and the presence of pyuria (>5 white blood cells/hpf), hematuria (>5 red blood cells/hpf), bacteriuria (with morphology), and crystalluria (with type[s]). Urine microbial culture and sensitivity testing results were reviewed for bacterial growth.

The location(s), number, and size of the uroliths were extracted from abdominal imaging data. Anatomic location was classified as kidney, ureter, bladder, or urethra. Uroliths noted at each anatomic location were counted and categorized on an ordinal scale as none, 1-3, 4-6, and >6 as per a previous publication.^5^ For dogs with radiographs, the maximum diameter of the largest urolith was recorded. If this data was not available in the radiologist’s report, it was extracted through review of radiographic images by one of the authors (E.F.). Serial imaging results were compared, and the timepoints were recorded for when partial dissolution (reduction in urolith diameter, number, or both) and complete dissolution (no residual uroliths) were first documented. In cases with urolith analysis, results were categorized as struvite (>70% struvite composition throughout all layers), mixed struvite (struvite present at ≤70% in the body, shell, or nidus), compound struvite (struvite present at >70% in at least 1 but not all layers), or non-struvite (>70% other mineral types throughout all layers).^12^

### Urolith composition prediction

Two tools, CALCulate and CALCurad, available in the free Minnesota Urolith Center application (MN Urolith, Hawthorne Mackenzie Digital; accessed July through November 2024), were retrospectively utilized by the authors to determine how results corresponded to dissolution outcomes. CALCulate reports data from the Minnesota Urolith Center on the composition of uroliths for specific signalments (Figure S1A-C). CALCurad uses a deep learning algorithm to predict if the radiographic appearance of a urolith is consistent with a struvite composition (Figure S1D-F). The accuracy of CALCurad alone is 81%,^13^ and the combined accuracy of these 2 tools is reported to be 96% for predicting struvite uroliths in dogs.^14^ In this study, the tools were used individually rather than in a stepwise manner. For CALCulate, each dog’s signalment (breed, sex, and age at the time of urolith diagnosis) was inputted with 2 outputs recorded: (1) the most common urolith composition for the signalment and (2) the percentage of uroliths composed of struvite for the signalment. The CALCurad input was a photograph of radiographic imaging of the urolith(s), and the output was dichotomous: “struvite” or “non-struvite.” CALCurad was only performed for cystoliths.

The radiographic appearance of cystoliths was additionally assessed by one of the authors (E.F.) for the following characteristics: pyramidal shape, ovoid shape, and maximum cystolith diameter ≥ 10 mm. Struvite constitutes 100%, 83%, and 93%, respectively, of uroliths with these features.^15^ Cystoliths were also evaluated for concentric rings of different radiopacity, suggestive of compound uroliths.^12^

### Outcomes and adverse events

Outcomes were categorized as complete dissolution of uroliths, partial dissolution (reduction in urolith diameter, number, or both), no dissolution (no reduction in urolith burden on the final imaging study after a minimum of 4 weeks of therapy), or undetermined (lack of reduction in urolith diameter after fewer than 4 weeks of therapy). The minimum requirement of 4 weeks for determining a no dissolution outcome was selected based on previous data from dogs with successful dissolution of suspected struvite cystoliths.^5^ While dissolution is not always noted by < 15 days, at least partial dissolution is apparent on radiographs by 15-28 days.^5^ Potential adverse events were recorded, such as persistent signs of lower urinary tract disease, urethral obstruction, or new onset of clinical signs of gastrointestinal disease (vomiting, diarrhea, or inappetence).

### Statistical analysis

Continuous variables were assessed using quantile-quantile plots and Shapiro–Wilk tests to determine if they followed a normal distribution. Those with a normal distribution are reported as mean ± SD; range is also provided for some to show the full spread of the data. Variables that did not follow a normal distribution are summarized as median (range); interquartile range (IQR) is also provided for some. Ordinal variables (body condition score [1-9 scale] and dipstick urine pH) are reported as median (range). Categorical variables are summarized as proportions where applicable. The primary study outcome was complete urolith dissolution, and the 95% confidence interval (CI) for the proportion of dogs achieving this outcome was calculated using the Wilson score interval. For dogs with both a baseline and final urinalysis, a Wilcoxon signed-rank test and a paired t-test were performed to compare urine pH and USG at baseline versus at the end of the dissolution trial, respectively. Statistical analyses were performed using R software for statistical computing (R, version 4.2.2).^16^  *P* values < .05 were considered statistically significant.

## Results

### Description of cases

The medical record query identified 21 dogs with suspected struvite uroliths that were fed the therapeutic multipurpose urinary food and received antimicrobial therapy or had documented absence of bacteriuria. Five dogs were excluded due to lack of follow-up imaging, 2 were excluded because they were switched to a different therapeutic urinary food before completion of the dissolution trial, and 1 dog was excluded for concurrent d,l-methionine therapy. The remaining 13 dogs were included in this study. Individual dog data is provided in Table S1. Of the 13 dogs, 12 were spayed females and 1 was a neutered male. The mean age was 6.4 ± 2.1 years, and the median weight was 11.3 kg (range 3.0-44.2). The median body condition score was 6.0 (range 5.0 to 7.5). Breeds represented included: Miniature Schnauzer (2), Cavalier King Charles Spaniel (2), mixed breed (2), and 1 each of Bichon Frise, Great Pyrenees, Labrador Retriever, Pug, Shih Tzu, Standard Poodle, and Vizsla. Six dogs had a history of 1 or more previous episodes of bacterial cystitis, and 2 of these had previous struvite uroliths removed via cystotomy (1 and 4 years prior to the current presentation).

Baseline signs of urinary tract disease were present in 10 dogs and included hematuria (6), pollakiuria (5), stranguria (4, 1 with urethral obstruction), urinary accidents (3), dribbling urine (2), and urinary urgency without accidents (1). Of dogs without urinary signs, 1 presented with anorexia and lethargy (diagnosed with a urethral obstruction), 1 with vomiting and shivering (diagnosed with a ureteral obstruction), and 1 with no current clinical signs but historic lower urinary tract signs. The dissolution trial was initiated by a small animal internal medicine specialty service in nine cases and by a primary care service in 4 cases.

### Baseline urinalysis and urine microbial culture results

One dog did not have a urinalysis or urine microbial culture performed before starting the dissolution trial. Of the 12 dogs with a baseline urinalysis, 4 were receiving antimicrobial therapy at the time. Detailed urinalysis results are provided in Table S1. The median baseline urine pH was 7.3 (range 6.0-8.5); it was alkaline in 6 cases, neutral in 4 cases, and acidic in 2 cases. The mean baseline USG was 1.030 ± 0.010. The urinalysis detected dipstick proteinuria in 11, hematuria in 8, and pyuria in 8 dogs. Cytologic bacteriuria was identified in 5 cases (all samples collected via cystocentesis) and was coccoid for all. Struvite crystalluria was reported in 5 dogs; no other crystal types were observed.

Urine microbial culture and sensitivity were performed at the baseline visit for nine dogs. Bacterial growth occurred in 4 (all in samples collected via cystocentesis) and included *Staphylococcus intermedius* (1), dual growth of *S. intermedius* and β-hemolytic *Streptococcus* spp. (1), coagulase-negative *Staphylococcus* spp. (1), and gram-positive cocci with mixed flora (1). All 5 dogs with no bacterial growth were either currently receiving antimicrobial therapy (3) or had received it within the past 30 days (2).

When both baseline and past visits were evaluated, 10 of the 13 dogs had cytologic or microbial bacteriuria noted on a urinalysis or urine microbial culture, respectively, within the last 60 days. Of the 3 dogs without bacteriuria noted, 1 was the dog without urinalysis or urine microbial culture data, and 2 had pyuria on the baseline urinalysis but lacked urine microbial culture.

### Urolith characteristics

Uroliths were identified by abdominal radiography in 12 dogs and by ultrasonography in 1 dog. Uroliths were located in the bladder only in 8 dogs, bladder and kidney in 2 dogs, bladder and urethra in 1 dog, urethra only in 1 dog, and kidney and ureter in 1 dog. The 2 dogs with urethroliths were obstructed at the baseline visit and had the urethroliths retropulsed into the bladder prior to the start of the dissolution trial. The dog with ureteroliths had a ureteral stent placed at the baseline visit. Two dogs had 1 to 3 uroliths, 4 had 4 to 6 uroliths, and seven had >6 uroliths. For the 12 dogs with radiographs, the mean of the maximum urolith diameter was 12.4 ± 5.1 mm (range 5.0-20 mm).

### Dissolution protocol

Eight dogs were fed a dry formulation of the therapeutic multipurpose urinary food, 1 was fed a canned formulation, and 4 were fed a combination of dry and canned. Three of the dogs had been fed the food historically, prior to the current urolith diagnosis; one of these dogs was also receiving other foods and herbal supplements (ingredients not available) until the trial started. The other 10 dogs were started on the urinary food at the time of or shortly after urolith diagnosis. Three dogs received treats during the dissolution trial (see *Urinary food adherence*).

Twelve of the 13 dogs received antimicrobial therapy during the dissolution trial. Antimicrobials prescribed included amoxicillin and clavulanic acid (7; Clavamox, Zoetis), amoxicillin (2), doxycycline (1), cephalexin followed by amoxicillin and clavulanic acid (1), and cefpodoxime proxetil (Simplicef, Zoetis) followed by amoxicillin (1). The mean duration of antimicrobial therapy was 63 ± 42 days (range 7-146). The remaining dog had a 10-day course of amoxicillin and clavulanic acid that ended 28 days prior to starting the urinary food; this dog had no bacterial growth on baseline urine microbial culture.

Two dogs received a 3-day course of carprofen. No other steroidal or non-steroidal anti-inflammatory medications were prescribed.

### Dissolution and clinical outcomes

All dogs had abdominal radiographs performed during follow up, and 1 dog additionally had a urinary tract ultrasound performed.

#### Complete dissolution

Complete dissolution of cystoliths was achieved in 9/13 dogs (69%, 95% CI, 42-87). This included 8 dogs with cystoliths (including the 2 with retropulsed urethroliths) and 1 dog with nephroliths and a ureterolith. Median time until repeat abdominal imaging was 35 days (range 15-60, IQR 25-43), with all achieving at least partial dissolution at that time. Mean time until documentation of complete dissolution was 71 ± 52 days (range 15-175; IQR 37-89) based on 8 dogs. The time until complete dissolution could not be determined for the ninth dog. This dog had a reduction in radiographic cystolith size at day 26 with residual “grainy material,” suspicious for <1 mm uroliths. The trial was continued for 2 more weeks, but the dog did not return for imaging until 10 months later at which time a urinary tract ultrasound showed no uroliths.

Two of the dogs with complete dissolution had analysis of uroliths cystoscopically retrieved prior to starting the dissolution trial. One of the dogs had a urethral obstruction on presentation with retropulsion of urethroliths into the bladder. Cystoscopy was then performed with several stones fragmented with laser lithotripsy; complete stone removal was not pursued due to a large stone burden (>20 cystoliths with a maximum urolith diameter of 8 mm on post-procedure radiographs). The other dog underwent cystoscopy for a suspected bladder mass noted on point-of-care ultrasound by the primary veterinarian; radiographs were not performed prior. Cystoscopy revealed numerous cystoliths; 3 were retrieved with a basket for analysis, and dissolution was selected for those remaining due to a large stone burden (>20 cystoliths with a maximum urolith diameter of 19 mm on post-procedure radiographs). In both cases, the urolith compositions were struvite (100% struvite in 1 and 95% struvite and 5% calcium phosphate carbonate [CPC] in the other). The other seven dogs with complete dissolution did not have stones analyzed.

Baseline clinical signs resolved in 8 dogs with complete urolith dissolution. The final dog did not have clinical signs at the baseline visit and remained subclinical throughout the trial.

#### Other dissolution outcomes

Of the 4 dogs without complete dissolution, 1 had partial dissolution, and 3 had no dissolution. The dog with partial dissolution had cystoliths with a decrease in size and number at day 20, but no further changes were noted at day 41. The dogs with no dissolution (1 with cystoliths only and 2 with both cystoliths and nephroliths) had either no change in urolith size or number (2) or increased size (1) at a median of 48 days (range 41-154). No dog had an undetermined outcome as trials exceeded 4 weeks for those without complete dissolution.

The dogs without complete dissolution subsequently underwent urolith removal procedures including cystotomy (2), lithotripsy (1), and voiding urohydropropulsion (1). Urolith composition was non-struvite in 1 dog with a 100% CaOx urolith (body and shell). In the other 3 dogs, uroliths were mixed composition with bodies of 45%-60% struvite and 40%-55% CPC and shells composed of 0%-20% struvite, 25%-80% CPC, and 0%-75% CaOx; stones from one dog also had a nidus of 60% struvite and 40% CPC.

Baseline clinical signs resolved in 2 dogs with no dissolution, despite persistence of uroliths. Another dog had initial resolution and then recurrence of urinary signs. The last dog had improvement (decreased frequency) but not resolution of urinary signs.

### Patient and urolith prediction data by dissolution outcome

Patient and urolith data summarized by dissolution outcome are provided in Table 1 (individual results in Table S1).

**Table 1:** Patient, urolith, and baseline urinalysis data for 13 dogs undergoing a medical dissolution trial for suspected struvite uroliths.

**Variable**	**Dissolution outcome**	
	**Complete (*n* = 9)**	**Partial or none (*n* = 4)**
**Sex**	8 FS, 1 MN	4 FS
**Age**	6.3 ± 1.7 years	6.6 ± 3.2 years
**CALCulate data**		
**% struvite**	73 ± 22%	67 ± 14%
**Top composition is struvite**	7/9	4/4
**Weight**	19.5 ± 12.6 kg	8.3 ± 4.2 kg
**Current or recent (within 60 days) bacteriuria**	8/9	2/3
**Baseline urinalysis data^a^**		
**Urine pH**	7.0 (6.0-8.0)	7.5 (6.5-8.5)
	1/9 acidic 4/9 neutral 4/9 alkaline	1/3 acidic 1/3 neutral 1/3 alkaline
**USG**	1.030 ± 0.011	1.028 ± 0.006
**Struvite crystalluria**	4/9	1/3
**Final urinalysis data^b^**		
**Urine pH**	6.5 (6.0-8.0) 5/7 acidic 0/7 neutral 2/7 alkaline	7.5 (6.5-7.5) 1/3 acidic 0/3 neutral 2/3 alkaline
**USG**	1.031 ± 0.015	1.027 ± 0.009
**Struvite crystalluria**	0/7	0/3
**Antimicrobial therapy duration**	66 ± 49 days^c^	57 ± 27 days
**Maximum urolith diameter**	12.2 ± 5.2 mm^d^	10.7 ± 6.0 mm
**Urolith number** ** 1-3** ** 4-6** ** >6**	2 3 4	0 1 3
**Urolith location** ** Bladder only** ** Kidney only** ** Both**	8^e^ 1 0	2 0 2
**CALCurad struvite prediction^f^**	5/6	1/4^g^
**Radiographic cystolith features predictive of struvite^f^**		
**Pyramidal shape**	2/6	0/4
**Ovoid shape**	2/6	0/4
**≥10 mm diameter**	5/6	1/4
**Any of the 3 features**	5/6	1/4
**Urolith analysis**	1: 100% struvite 1: 95% struvite, 5% CPC 7: Not analyzed	1: 100% CaOx 1: body 60% struvite, 40% CPC; shell 75% CaOx, 25% CPC 1: body 45% struvite, 55% CPC; shell 10% struvite, 20% CaOx, 70% CPC 1: nidus 60% struvite, 40% CPC; body 35% struvite, 65% CPC; shell 20% struvite, 80% CPC

^a^One dog in the group without complete dissolution did not have a baseline urinalysis performed.

^b^Two dogs in the group with and one without complete dissolution did not have a final urinalysis performed.

^c^One dog in this group did not receive antimicrobial therapy during the dissolution trial.

^d^One dog was not included here as radiographs were not available to measure uroliths.

^e^Two dogs had urethroliths retropulsed into the bladder prior to starting the dissolution trial.

^f^CALCurad predictions and radiographic cystolith features could not be determined for 3 dogs in the complete dissolution group due to nephroliths only (1), urethroliths only (1), or no radiographs available (1). For the dog that underwent lithotripsy, CALCurad prediction and the 3 features predictive of struvite were performed on the pre-procedure radiographs, whereas the maximum urolith diameter, number, and location were determined using the post-procedure radiographs.

^g^The dog with a struvite CALCurad prediction in this group had partial dissolution noted. Results are separated by urolith dissolution outcomes, and continuous variables are reported as mean ± SD or median (range) depending on the data distribution. Abbreviations: CaOx = calcium oxalate; CPC = calcium phosphate carbonate; FS = female spayed; MN = male neutered; USG = urine specific gravity.

#### CALCulate output

The mean percentage of uroliths composed of struvite for the study dog signalments was similar between dogs with and without complete dissolution at 73 ± 22% (range 31%-95%) and 67 ± 14% (range 41%-84%), respectively. Struvite was the most common composition based on signalment for seven of nine dogs in the group with complete dissolution and all dogs without complete dissolution.

#### Urinalysis and urine microbial culture data

Urinalyses and urine microbial culture results were available for 10 and 9 dogs, respectively, at the final visit. Of those with complete dissolution, 1/9 had an acidic urine pH at baseline versus 5/7 at the final visit. Of those without complete dissolution, 1/3 had an acidic urine pH at the baseline and final visits. Across dogs in both groups with paired baseline and final urinalyses, the median urine pH at the final visit (6.5, range 6.0-8.0) was lower than at the baseline visit (7.5, range 6.5-8.5; *P* = .041). The final visit mean USG for these dogs was 1.028 ± 0.012, which did not represent a statistically significant change from baseline (1.031 ± 0.008; *P* = .50).

Of the seven dogs with complete dissolution and a final urinalysis, none had cytologic bacteriuria or pyuria; 6 also had urine microbial cultures with no bacterial growth detected. Of 3 dogs with no dissolution and a final urinalysis, 1 had persistent pyuria and coccoid bacteriuria with growth of coagulase negative *Staphylococcus* spp. on culture; this was one of the dogs with mixed composition struvite CPC uroliths.

#### CALCurad results and other urolith features

**Figure 1:**
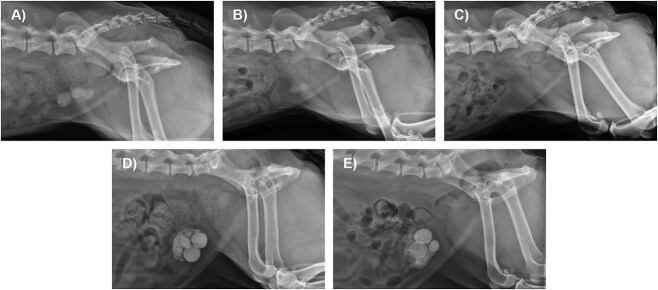
Lateral radiographs of 2 dogs undergoing medical dissolution for suspected struvite cystoliths using a therapeutic multipurpose urinary diet and antimicrobial therapy. Panels (A-C) are right lateral projections for a 5-year-old female spayed Shih Tzu with complete dissolution of cystoliths. (A) At the baseline visit, greater than 10 cystoliths were visible that measured up to 15 mm in diameter with at least 3 having an ovoid shape. (B) On day 43, significant dissolution was noted with 2 cystoliths remaining. (C) By day 85, no cystoliths were detected. Panels D and E are left lateral projections for a 10-year-old female spayed Bichon Frise with no dissolution of cystoliths. (D) At the baseline visit, greater than 10 cystoliths were visible that measured up to 19 mm in diameter. (E) On day 50, there was no radiographic evidence of cystolith dissolution. Concentric rings of varying radiopacity are visible on the 2 largest cystoliths. Urolith analysis revealed a mixed composition: a nidus of 60% struvite and 40% calcium phosphate carbonate (CPC), body of 35% struvite and 65% CPC, and a shell of 20% struvite and 80% CPC.

The CALCurad tool was run for the 10 dogs with radiographically detected cystoliths. All dogs with a CALCurad prediction of struvite uroliths had complete (5) or partial (1) dissolution; complete dissolution also occurred in 1 dog that CALCurad predicted had non-struvite uroliths. Six dogs had radiographic cystolith features predictive of a struvite composition, including a pyramidal shape (2, Figure S1E), ovoid shape (2, Figure 1A), and maximum cystolith diameter ≥ 10 mm (6). Five of the dogs with these cystolith features had complete dissolution. The exception was the only dog that had cystoliths with concentric rings of varying radiopacity, suggestive of a compound composition. Example radiographs for this dog and a dog with complete dissolution are provided in Figure 1.

#### Urinary food adherence

Two dogs with complete dissolution were fed “occasional” non-urinary treats, including human foods (1) and bones (1). Both had an acidic final urine pH. Another dog with no dissolution was fed urinary treats from a different manufacturer (ROYAL CANIN Urinary Canine Treats, Royal Canin). This dog had an alkaline final urine pH but was also the dog with *Staphylococcus* spp. growth on final urine microbial culture.

#### Antimicrobial therapy

Eight of the nine dogs in the complete dissolution group received antimicrobial therapy during the trial for a mean of 66 ± 49 days (range 7-146). This included the 2 dogs being exclusively fed the urinary diet prior to the baseline visit (both of which had baseline bacteriuria). The complete dissolution group also included the 1 dog that received the urinary food without concurrent antimicrobial therapy (absence of baseline bacteriuria confirmed after antimicrobial therapy). Of dogs without complete dissolution, all 4 received antimicrobial therapy for a mean of 57 ± 27 days (range 41-97).

### Adverse events

Two dogs experienced gastrointestinal signs during the trial, including a single episode of vomiting (1) and transient inappetence (1). The dog with vomiting had a history of chronic vomiting prior to being fed the study food. No dog developed a urethral obstruction during the dissolution trial.

## Discussion

In this retrospective study, feeding a therapeutic multipurpose urinary food (formulated for both struvite dissolution and CaOx prevention) with concurrent or recent antimicrobial therapy effectively dissolved struvite uroliths in dogs. All dogs with only partial or no dissolution had uroliths with non-struvite mineral shells. No dog developed a urethral obstruction during the dissolution trial, despite 2 dogs with obstructions on presentation. These results support the recommendation that medical dissolution should be attempted for suspected struvite uroliths in dogs and strengthen the evidence that eradication of bacteriuria plus feeding of a therapeutic multipurpose urinary food are effective for this purpose.

In this study, the proportion of dogs with complete dissolution was 9/13 (69%). Protocols using antimicrobial therapy and other therapeutic urinary foods have success rates of 5/10 (50%) and 28/41 (68%) for dissolution of suspected struvite cystoliths,^5^^,^^9^ and the success rate of a protocol using antimicrobial therapy and a urinary acidifier without a therapeutic urinary food is 8/11 (73%).^11^ Since the primary reason for lack of urolith dissolution in all studies was a non-struvite composition,^5^^,^^9^^,^^11^ differences in the success rates are likely due to random sampling error. Medical dissolution of struvite nephroliths using antimicrobial and dietary therapy is also effective in dogs,^10^ and it was documented in 1 case here. In contrast, dissolution of struvite uroliths with antimicrobial therapy alone (fed non-urinary foods) is uncommonly described.^3^^,^^17^ Data on struvite urolith dissolution with therapeutic urinary food alone, without antimicrobial therapy, is lacking in dogs with spontaneous urinary tract infections. Here, 2 dogs were exclusively fed the therapeutic urinary food prior to urolith diagnosis; these dogs went on to have complete dissolution after receiving antimicrobial therapy for urease-producing bacteriuria. Together, the data support the effectiveness of therapeutic urinary food or urinary acidifier combined with antimicrobial therapy for successful dissolution of struvite cystoliths and nephroliths in dogs.

Mean time to documentation of complete dissolution was 71 days in this study, compared to 31-35 days in previous studies.^5^^,^^9^ There are a few points to consider when interpreting the current study’s dissolution time. First, the time represents when dissolution was documented with medical imaging, not when it first occurred. Several dogs did not return for follow-up imaging until 2 or more months after their previous visit, likely leading to an overestimation of the time it took to achieve dissolution. Second, dissolution time might be affected by the size of the uroliths. In this study, the mean of the maximum urolith diameter was 12 mm in dogs with successful dissolution. By comparison, a previous study reported that the maximum urolith diameter had a median of 6 mm in dogs with successful dissolution.^5^ Differences in dissolution protocols (food type, antimicrobial selection, or duration) might also have contributed. Finally, given the small size of the study, differences might be due to random sampling error.

While ≤ 10% of a non-struvite mineral might not prevent complete dissolution,^5^ greater proportions are a common reason for dissolution failure.^5^^,^^9^^,^^11^ Here, all cases without successful dissolution had urolith shells containing a predominantly calcium component (CaOx, CPC, or both). Distinguishing struvite from non-struvite or mixed/compound uroliths is challenging, even in prospective trials.^9^^,^^11^ Here, we retrospectively used the MN Urolith application CALCulate and CALCurad tools to aid in prediction of urolith composition. The combined accuracy of these tools is 96% for struvite uroliths in dogs.^14^ While the mean CALCulate output for struvite prevalence was similar between dogs with and without complete dissolution (73% and 67%, respectively), both were higher than the observed struvite prevalence for uroliths obtained from dogs across the United States (41%).^1^ The CALCurad predicted struvite for 6 dogs in this study, all of which had some degree of dissolution (5 complete and 1 partial); only 1 dog with complete dissolution had a non-struvite prediction. Though study numbers are small, results align with the reported CALCurad accuracy of 81%.^13^Since urease-producing bacteriuria and alkaline urine are also consistent with struvite uroliths,^3^ we evaluated urinalysis and urine microbial culture results. Eight of 9 dogs with complete dissolution had current or recent bacteriuria. The median urine pH at the baseline visit was alkaline, but some dogs had an acidic or neutral urine pH; these results were likely confounded by current or recent antimicrobial therapy. Due to the retrospective nature of this study, it is not possible to determine how clinicians used signalment, radiographic urolith appearance, urinalysis, and urine microbial culture findings to guide their decision to pursue a dissolution trial. Overall, the results show that successful urolith dissolution is likely in populations where these data support a struvite composition, but that dissolution can also occur in some dogs with inconsistent data.

The dogs in this study generally had a long duration of antimicrobial therapy with a mean of 66 days in those with complete dissolution. In a similar retrospective study, the duration was 30 days for dogs that achieved cystolith dissolution.^5^ In prospective studies, antimicrobial therapy was given until complete dissolution was achieved or for 2 weeks beyond dissolution.^9^^,^^11^ While the International Society for Companion Animal Infectious Diseases has proposed short courses (7 days) of antimicrobial therapy for struvite urolith dissolution,^18^ this was based on data showing resolution of struvite crystalluria rather than actual uroliths in dogs.^19^ Here, 1 dog received a 7-day course of antimicrobial therapy with complete urolith dissolution by day 15. An additional dog had complete dissolution without concurrent antimicrobial therapy but had no baseline bacteriuria on urine microbial culture. More data is needed to determine the optimal duration of antimicrobial therapy for struvite urolith dissolution in dogs.

As expected, most dogs had an acidic final urine pH. Acidic urine is an indication that urease-producing bacteriuria resolved and that the food is acidifying. Urinary pH has a major impact on struvite solubility, with increasing dissolution rates at more acidic values.^20^ If alkaline urine is noted during a dissolution trial, a urine microbial culture and confirmation of food adherence should be checked. In this study, 3 dogs had an alkaline final urine pH. One had no dissolution with persistent urease-producing bacteriuria and feeding of nonurinary treats. However, the role these played in dissolution failure is unclear, as the dog’s uroliths had a calcium component. The reason for the alkaline urine pH in the 2 dogs with complete urolith dissolution was undetermined. It might indicate unrecorded consumption of nonurinary foods or reflect a postprandial alkaline tide.^21^

Concentration of minerals in the urine also affects struvite solubility.^22^ Increasing water excretion is therefore expected to promote dissolution. Most dogs were eating a dry formulation of the study food, and the USG values did not differ from the baseline to the final visit in this study. Feeding of a different therapeutic multipurpose urinary food as part of a struvite dissolution trial increased USG.^9^ These data suggest that lowering USG is not necessary to achieve struvite urolith dissolution in dogs. A limitation of this interpretation is that values obtained through single point samples at the hospital might not reflect daily averages or water consumption at home.^23^^,^^24^

No urethral obstructions occurred during the dissolution trials in this study. The study included 2 dogs with urethral obstructions on presentation that were relieved via retropulsion; both of these dogs achieved complete dissolution. Despite the lack of reobstruction in these dogs, urethral obstructions are considered a contraindication to medical dissolution based on previous data and expert consensus.^4^^,^^5^ Here, the only adverse events noted were self-limiting gastrointestinal signs in 2 dogs, 1 of which had historic gastrointestinal signs.

A limitation of this study is its small size. Prior to the study timeframe, there was a therapeutic food primarily formulated for struvite urolith dissolution. This food was the standard for medical dissolution of struvite uroliths at our hospital until it went off the market. Study case numbers were likely also low due to the referral nature of our hospital; most dogs with struvite uroliths are presumably managed by their primary veterinarian. The retrospective nature of this study further creates limitations; there was no standardization for the timing of follow-up imaging, making it difficult to accurately estimate time to dissolution.

### Conclusion and clinical importance

In summary, feeding a therapeutic multipurpose urinary food is effective as the nutritional component of a struvite urolith dissolution protocol. Current or recent bacteriuria, an alkaline urine pH, an at-risk signalment, and a consistent radiographic appearance of uroliths were common features of dogs with successful dissolution. A non-struvite composition of 1 or more urolith layers should be suspected in dogs with minimal to no dissolution despite appropriate nutritional intervention and antimicrobial therapy.

## Supplementary Material

aalaf061_Supplemental_Files

## Off-label antimicrobial declaration

This manuscript describes extra-label use of antimicrobials with duration extending beyond what is listed on the product labels. It also reports extra-label use of human antimicrobials in dogs.

## Institutional animal care and use committee or other approval declaration

Authors declare no institutional animal care and use committee or other approval was needed.

## Abbreviations

ACVIM: American College of Veterinary Internal Medicine
CaOx: calcium oxalate
CPC: calcium phosphate carbonate
UMN VMC: University of Minnesota Veterinary Medical Center
USG: urine specific gravity
