# Supplementary figures and images for: Outcomes of medical dissolution for suspected struvite uroliths in dogs using a therapeutic multipurpose urinary diet and antimicrobial therapy
Source: J Vet Intern Med. 2026 Jan 21;40(1):aalaf061. doi: 10.1093/jvimsj/aalaf061 (PMC12881935; doi:10.1093/jvimsj/aalaf061)

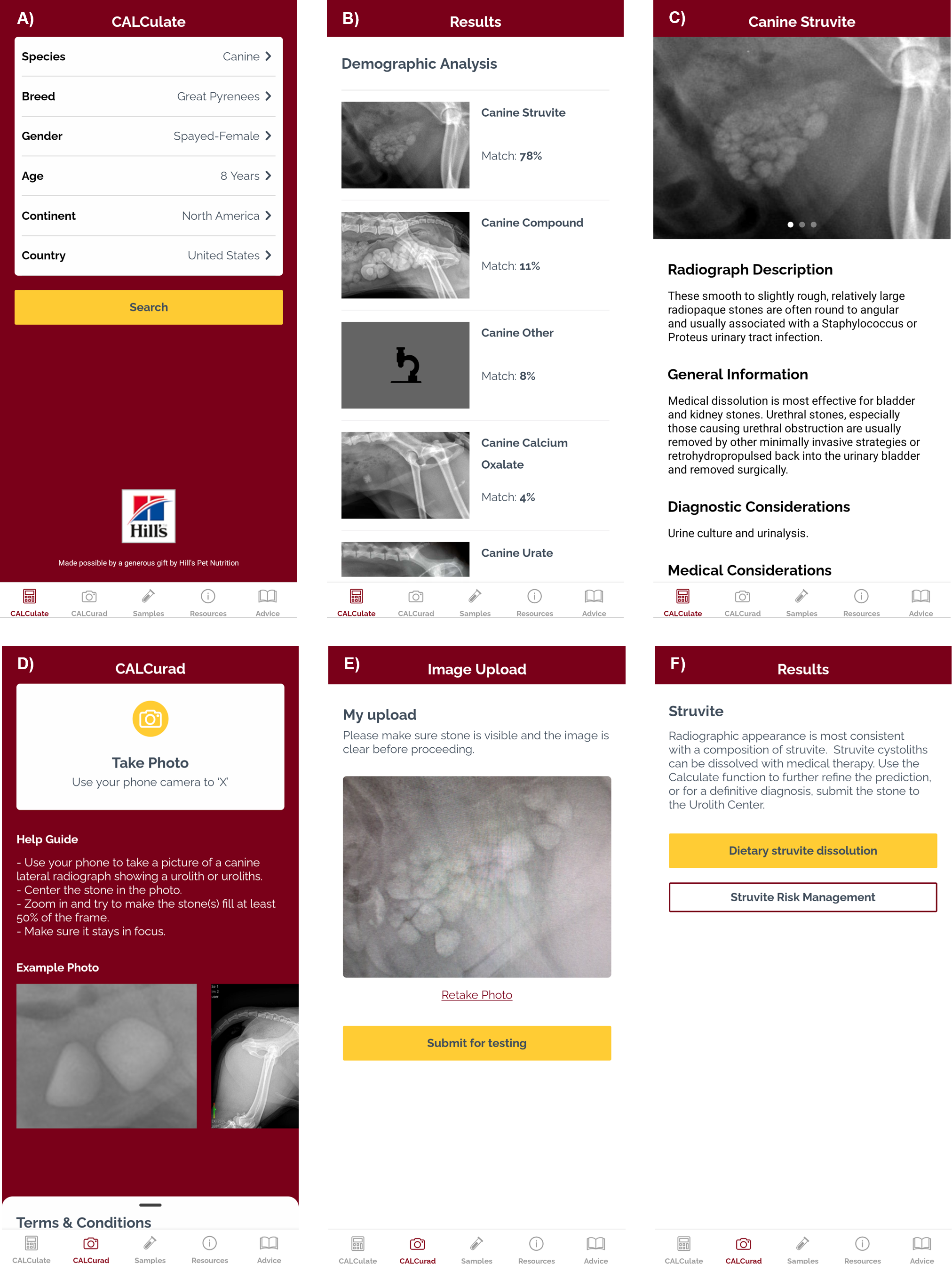

Supplement: aalaf061_Supplemental_Files [file aalaf061_supplemental_files.zip › Struvite_Dissolution_Supplementary_Figure1_aalaf061(1).png]
